# The effects of walking intervention on preventing neck pain in office workers: A randomized controlled trial

**DOI:** 10.1002/1348-9585.12106

**Published:** 2019-12-18

**Authors:** Ekalak Sitthipornvorakul, Rattaporn Sihawong, Pooriput Waongenngarm, Prawit Janwantanakul

**Affiliations:** ^1^ Department of Physical Therapy Faculty of Allied Health Sciences Chulalongkorn University Bangkok Thailand

**Keywords:** disability, exercise, musculoskeletal disorder, sedentary worker

## Abstract

**Objective:**

This study aimed to evaluate the efficacy of increased daily walking steps on the 6‐month incidence of neck pain among office workers.

**Methods:**

Healthy office workers with high risk of neck pain were recruited into a 6‐month prospective cluster‐randomized controlled trial. Participants were randomly assigned at the cluster level, into either intervention (n = 50) or control (n = 41) groups. Participants in the intervention group were instructed to increase their daily walking steps to a designated level for a duration of 6 months. Participants in the control group received no intervention. The outcome measures included the 6‐month incidence of neck pain as well as its pain intensity and disability level. Analyses were performed using multivariable logistic regression model.

**Results:**

Of the participants in the intervention and control groups, 22% and 34% reported a 6‐month incidence of neck pain, respectively. After adjusting for confounders, a significant preventive effect of walking intervention was found (adjusted odd ratio 0.22, 95% confidence interval 0.06‐0.75). No significant difference in pain intensity and disability level was found between those in the intervention and control groups.

**Conclusion:**

An intervention to increase daily walking steps reduced onset neck pain in high‐risk office workers. However, the walking interventions did not decrease pain intensity and disability in those increasing the number of daily walking steps compared to the control group.

## 1 INTRODUCTION

Neck pain is one of the most important musculoskeletal problems in office workers with 42%‐69% of office workers reporting neck pain and 34%‐49% of office workers developing new onset of neck pain every year.^1^, ^2^, ^3^, ^4^ Although rapid improvement in neck symptoms is common,^5^ 17% of office workers who report a new onset of neck pain developed chronicity.^6^ Neck pain causes personal suffering, disability, and impaired quality of work and life in general, which contributes to a great socioeconomic burden.^1^, ^7^, ^8^ Subsequently, the Neck Pain Task Force has called for effective strategies to prevent neck pain.^1^

A recent systematic review of randomized controlled trials revealed moderate‐quality evidence supporting the effectiveness of an exercise program for reducing the risk of a new episode of neck pain.^7^ However, exercise adherence is paramount to the efficacy of exercise intervention^9^, ^10^ and a previous study showed that adherence to an exercise program to prevent neck pain among office workers was only low to moderate during 1‐year follow‐up.^11^ Walking, which is a fundamental human activity, has been found to improve exercise adherence compared to supervised exercise programs.^12^, ^13^ A 1‐year prospective cohort study in healthy sedentary workers reported a significant negative association between daily walking steps and the onset of neck pain.^14^ There has been no randomized controlled trial investigating the efficacy of walking intervention in preventing neck pain among office workers. Therefore, the objective of this study was to evaluate the effect of increased daily walking steps on the 6‐month incidence of neck pain among office workers. It was hypothesized that a group of office workers receiving the walking intervention program would experience a reduction in onset neck pain compared to a group of office workers receiving no intervention.

## 2 MATERIALS AND METHODS

### 2.1 Study population and procedures

A two‐armed, parallel‐group, cluster randomized controlled trial with 6‐month follow‐up was conducted in a convenience sample of office workers recruited from 4 enterprises in Bangkok. The study was approved by the University Human Ethics Committee and was registered in the Thai Clinical Trials Registry (TCTR20160928001). The participating enterprises were three government offices and a public university office. The inclusion criteria were individuals aged between 18 and 55 years, working full‐time, having at least 1 year of experience in the current position, and at risk of nonspecific neck pain evaluated by the Neck Pain Risk Score for Office Workers (score ≥2).^15^ The exclusion criteria were individuals having neck pain in the last 12 months with pain intensity >30 mm on a 100‐mm visual analogue scale,^14^, ^16^ reporting pregnancy or having planned to become pregnant in the next 6 months, having a history of trauma or accidents in the spinal region, or having a history of spinal, intra‐abdominal and femoral surgery in the last 12 months. Subjects who had been diagnosed with infection of the spine and discs, ankylosing spondylitis, congenital anomaly of the spine, spondylolisthesis, tumor, spondylosis, systemic lupus erythymatosus, rheumatoid arthritis, or osteoporosis were also excluded.

Office workers who expressed interest completed a short screening questionnaire. If eligible, they were informed about the content and purpose of the project and gave their written informed consent to participate in the study. They were then asked to complete a baseline questionnaire and were randomly assigned at the cluster level into either the intervention or the control group using computer‐generated randomization software (http://www.randomizer.org) with an allocation ratio of 1:1 by a researcher (ES). Clusters of participants were located in the same workplaces to avoid contamination of the intervention and to enhance compliance within the intervention group.^17^ A total of 4 clusters (2 clusters for the intervention group and 2 clusters for the control group) were identified and the cluster size ranged from 17 to 32 participants.

### 2.2 Questionnaire

Individual, work‐related physical, and psychosocial characteristics of participants were collected at baseline. Individual factors included age, gender, body weight, height, marital status, level of education, frequency of exercise or sport, smoking habits, and number of driving hours per day. Work‐related physical factors included years of working experience, number of working hours, and current job position. Respondents were asked about the duration of working with a computer, rest breaks, and performing various activities during work. They were asked to self‐rate the ergonomics of their workstations (table, chair and monitor position) and work environment conditions (ambient temperature, noise level, light intensity and air circulation). The Job Content Questionnaire (Thai version) was used for measuring the psychosocial factors.^18^

### 2.3 Interventions

Participants in the intervention group were asked to increase their daily walking steps to a designated level for a duration of 6 months. The designated daily walking steps for each participant in the intervention group was calculated based on data from a 1‐year prospective cohort study of the association between daily walking steps and incidence of neck pain in office workers.^14^ First, multiple linear regression analysis was performed on data from office workers in the previous study who reported no incident neck pain during 1‐year follow‐up to identify a set of factors that can optimally predict their daily walking steps. Second, these identified factors (ie, gender, age, body weight, height, history of neck pain, sitting ≥2 hours and standing ≥2 hours) were used to build a mathematic formula to calculate the number of daily working steps required to prevent neck pain in office workers. Third, we developed a smartphone application with the mathematic formula embedded. Each participant was asked to install the application in their smartphone and provided their personal information in order to obtain their designated daily walking steps. Participants in the control group received no intervention.

Data on daily walking steps were collected every day using the smartphone application over a 6‐month period. Participants were instructed to carry the smart phone with application in their pocket, from getting up in the morning until going back to bed at night. Results regarding the reliability and validity of using the smartphone application to collect daily walking steps among office workers have been reported elsewhere.^19^ Both intervention and control groups received financial incentives every month until completing the 6‐month follow‐up or withdrawing from the study. For the intervention group, participants received incentives progressively, according to the number of days per month that they achieved their designated daily walking steps. For the control group, participants received the same incentive compensation every month. No other recommendation on how to increase daily walking steps was provided to participants in both groups. During the study, participants in both groups were requested to keep the level of their physical activity unchanged.

### 2.4 Outcome measures

Incidence of nonspecific neck pain, which is neck pain (with or without radiation) without any specific systematic disease being detected as the underlying cause of the complaints,^20^ during the 6‐month follow‐up was evaluated. In this study, cases were defined as those who answered “Yes” to the question “Have you experienced any neck pain lasting >24 hours in the past month?”, reported pain intensity >30 mm on a 100‐mm visual analogue scale, and had no numbness or weakness in the upper limbs. The body chart of the standardized Nordic questionnaire was used to define the area of the neck.^21^ Disability related to neck pain, as measured by the Neck Disability Index (NDI),^22^ was also assessed. A diary was used to collect all health outcomes. The researcher returned to collect the diaries from participants every month until completing the 6‐month follow‐up or withdrawing from the study.

### 2.5 Statistical analysis

The analysis followed an intention‐to‐treat approach. Comparisons of the baseline characteristics of participants between intervention and control groups were conducted using the independent *t* test for continuous data and Chi‐square for nominal and ordinal data. The incidence rate of neck pain was calculated for each group. The study was designed to have 80% power to show a 10% difference in the incidence rate of neck pain at the one‐sided 5% level between those who received and did not receive the walking intervention. The incidence of neck pain was expected to be 16.0%.^14^

A multivariable logistic regression model was built to assess the effect of walking intervention on the incidence of neck pain. All 50 possible covariates were assessed by entering potential covariates into the logistic regression model one at a time. The unadjusted and adjusted odd ratios (ORs) were then compared. The final logistic regression model included covariates that altered the unadjusted and adjusted OR by at least 10%.^23^ The unadjusted and adjusted ORs and 95% confidence interval (CI) for the final model are presented in the results.

Health outcomes, that is, pain intensity and disability, between those reporting neck pain, regardless of pain intensity, in the intervention and control groups were compared using independent t‐tests. Statistical significance was set at the 5% level. All statistical analyses were performed using SPSS statistical software, version 24.0 (SPSS Inc).

## 3 RESULTS

**Figure 1:**
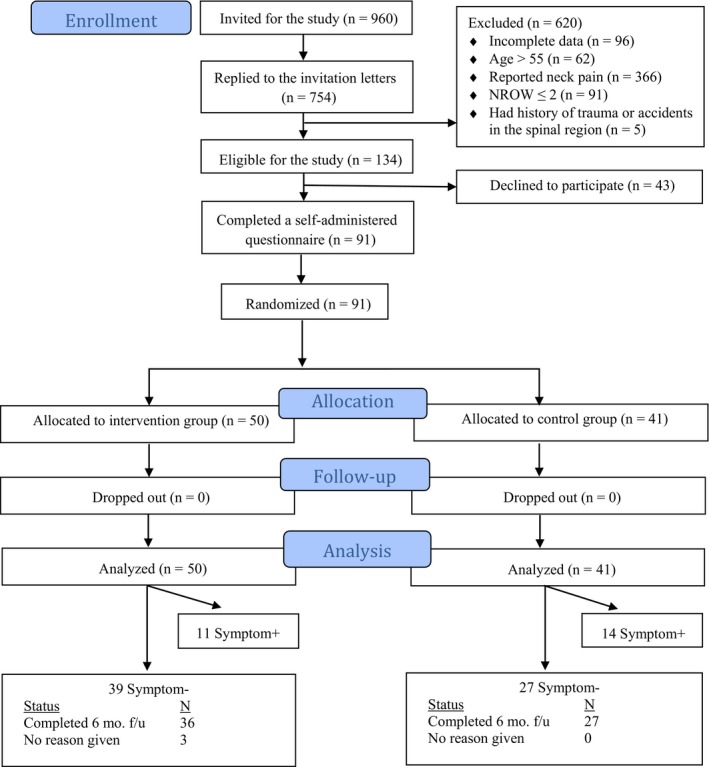
CONSORT flowchart of the study

The flow of participants is shown in Figure 1. The randomization was relatively successful in creating the intervention and control groups with similar baseline characteristics (Table 1). The only exceptions were the measures of age and duration of employment. The mean (SD) designated walking steps calculated by the smartphone application for participants in the intervention group was 7735 (3094) steps per day. During the 6‐month follow‐up, the mean (SD) of adherence to the designated daily walking steps among office workers in the intervention group was 20.2 (10.1) days per month, accounting for 71% of full adherence (Table 2). Mean daily walking steps for participants in the intervention group was higher than for those in the control group (Table 3).

**Table 1:** Baseline characteristics of participants

Characteristic	Mean (SD)		*P* value
	Intervention group (n = 50)	Control group (n = 41)	
Demographic characteristics			
Age (years)	35.0 (8.7)	30.3 (4.9)	.003*
Gender: female (%)	76	78	.817
Weight (kg)	59.3 (13.7)	59.4 (11.8)	.969
Height (cm)	160.6 (7.1)	163.2 (8.6)	.133
Body mass index (kg/m^2^)	22.86 (4.24)	22.19 (2.84)	.519
Education (%)			.570
Lower than Bachelor's degree	10.0	9.8	
Bachelor's degree	68.0	58.5	
Higher than Bachelor's degree	22.0	31.7	
Exercise frequency in the past 12 mo (%)			.300
Never	12.0	24.4	
Occasionally	62.0	56.1	
Regularly	24.0	14.6	
Not sure	2.0	4.9	
Occupational‐related characteristics			
Duration of employment (years)	10.1 (8.7)	5.3 (3.7)	.002*
Working hours per day (hours per day)	8.0 (0.8)	7.7 (1.1)	.115
Working days per week (days per week)	5.0 (0.1)	5.0 (0.3)	.926
Psychosocial characteristics			
Job control	35.7 (4.5)	36.2 (4.9)	.612
Psychological job demand	33.4 (4.8)	33.6 (4.1)	.857
Physical job demand	12.9 (3.0)	13.6 (2.8)	.296
Job security	16.6 (1.4)	16.3 (1.5)	.442
Social support	30.1 (4.0)	32.0 (5.6)	.054
Hazards at work	16.3 (3.6)	15.3 (2.8)	.144

Abbreviation: VAS, visual analogue scale.

* *P* value < 0.05 (independent *t* test).

**Table 2:** Mean number of days in which daily walking steps exceeded the designated daily walking steps per month and adherence percentage ratios among office workers in the intervention group (n = 50)

Follow‐up	Mean (SD) (days per month)	%
1st month	17.9 (10.8)	64
2nd month	20.1 (10.6)	71
3rd month	21.5 (9.9)	73
4th month	20.8 (10.1)	71
5th month	20.7 (9.4)	71
6th month	20.3 (9.6)	71
6‐month period	20.2 (10.1)	71

**Table 3:** Daily walking steps during 6‐month follow‐up for the intervention and control groups

Follow‐up	Mean (SD) (steps per day)		*P* value
	Intervention group (n = 50)	Control group (n = 41)	
1st month	7888 (2731)	7207 (2372)	.207
2nd month	8309 (3297)	7216 (2269)	.075
3rd month	8313 (3138)	7075 (2350)	.034
4th month	8286 (3256)	7126 (2477)	.057
5th month	8274 (3191)	6804 (2380)	.014
6th month	8072 (3051)	6834 (2273)	.029
6‐month period	8190 (3094)	7044 (2336)	<.001

Overall, 22% (11/50) of participants in the intervention group and 34% (14/41) of those in the control group reported incidence of neck pain during the 6‐month follow‐up. No harm or unintended effect was reported in either group.

Covariates were selected for multivariate logistic regression analysis, including age, gender, body mass index, duration of employment, and job security (Table 4). Multivariate logistic regression analysis revealed a significant effect of group assignment (walking intervention) on onset neck pain (adjusted OR 0.22, 95% CI 0.06‐0.75). The comparisons of pain intensity and disability level due to neck pain between the intervention and control groups indicated no significant difference (Table 5).

**Table 4:** Odd ratio (OR) and 95% confidence interval (CI) of the effects of walking intervention on incident neck pain (n = 91)

	Unadjusted OR (95% CI)	*P* value	Adjusted^a^ OR (95% CI)	*P* value
Group assignment (walking intervention)				
Control group	1.00		1.00	
Intervention group	0.48 (0.19‐1.24)	.131	0.22 (0.06‐0.75)	.016*
Age	1.05 (0.99‐1.11)	.119	0.99 (0.83‐1.17)	.862
Gender				
Male	1.00		1.00	
Female	2.57 (0.68‐9.67)	.162	4.23 (0.83‐21.49)	.082
BMI	1.15 (1.01‐1.31)	.032	1.20 (1.02‐1.38)	.023*
Duration of employment	1.05 (0.99‐1.12)	.113	1.09 (0.91‐1.330)	.342
Job security	0.71 (0.51‐0.98)	.035	0.67 (0.47‐0.97)	.035*

a All OR associated with particular covariates were adjusted for the effect of all other covariates that were in the model.

* *P* value < .05.

**Table 5:** Pain intensity and disability of participants reporting neck pain, regardless of pain intensity, during 6‐month follow‐up

Variable	Mean ± SD		*P* value
	Intervention group	Control group	
Pain intensity measured by VAS	1.2 ± 0.9 (n = 20)	1.4 ± 1.3 (n = 25)	0.597
Disability measured by NDI	2.8 ± 2.0 (n = 18)^a^	2.7 ± 2.8 (n = 24)^a^	0.856

Abbreviations: NDI, Neck disability index; VAS, visual analogue scale.

a There were 2 and 1 values of missing data in the intervention and control group, respectively.

## 4 DISCUSSION

The efficacy of walking intervention to prevent nonspecific neck pain among office workers was evaluated in this study. This randomized controlled trial showed that the walking intervention used in this study can reduce the 6‐month incidence rate of neck pain among high‐risk healthy office workers, who were identified using the neck pain risk score for office workers (NROW ≥2),^15^ by 78%. However, the walking intervention did not reduce pain intensity or disability level related to the neck pain of those increasing the number of daily walking steps compared to the control group.

A previous study reported daily walking steps in healthy office workers as ranging from 7602 to 8108 steps.^14^ The mean daily walking steps in those who received no walking intervention in the present study (7044 steps) were lower than the previous study. The discrepancy between the studies may be due to the inclusion criteria of the studied population. A specific group of healthy participants was selected for the present study, that is, those with high risk of neck pain as evaluated by the NROW^15^ to ensure that participants most in need were included. This risk score for predicting neck pain contains three questions regarding history of neck pain, chair adjustability, and perceived muscular tension. The tool has reasonable sensitivity, specificity, positive predictive value, and negative predictive value and is recommended for use in identifying those in need of early intervention.^15^ A previous 1‐year prospective cohort study reported a negative association between daily walking steps and the onset of neck pain.^14^ Thus, one plausible reason for lower daily walking steps among participants in the present study compared to the previous study relates to the fact that participants in the present study were at higher risk of neck pain than those in the previous study.

Exercise adherence is paramount to the efficacy of exercise intervention.^9^, ^10^ Previous studies investigating the efficacy of exercise programs in preventing or treating neck pain reported low to moderate participant adherence to the exercise programs (30%‐57%).^11^, ^24^ Findings have shown walking exercise to be associated with good participant adherence (80%).^13^ In this study, adherence to the designated daily walking steps among participants in the intervention group was also good (71%). This may be partly attributed to the financial incentives provided to this group's participants both monthly and progressively, according to the number of days per month that participants achieved their designated daily walking steps. On the other hand, participants in the control group received the same incentive compensation every month. Thus, based on the findings, providing regular and outcome‐based financial incentives proved effective in increasing participant adherence to a certain extent.

The present study is among the first of its kind to investigate the efficacy of a walking intervention to prevent nonspecific neck pain among office workers. Previously, Shnayderman et al^25^ found that a walk training program performed on a treadmill at low to moderate intensity was as effective as active movements combined with strengthening exercises for the trunk and upper/lower limbs on improving pain and disability in LBP patients. Recently, systematic review and meta‐analysis studies revealed that a walking intervention is an effective intervention in reducing pain and disability as well as improving quality of life when compared with other non‐pharmacological interventions in chronic LBP patients.^26^, ^27^ Our findings showed that the daily walking steps for the intervention group were greater than those for the control group in each month during the 6‐month follow‐up. However, the difference in the number of walking steps per day between the intervention (8190 steps per day) and control (7044 steps per day) groups was relatively small (approximately 1100 steps per day). Physical activity engagement differs across occupational categories. White‐collar workers, such as office workers, spend most of their time at work sitting and performing light occupational activities. They are most likely to have a low level of daily walking steps and therefore most likely to benefit from the walking intervention. The findings suggest that a small increase in a number of walking steps per day is sufficient to provide health benefits for them. The results are in line with the findings of a previous study showing that increasing daily walking steps by 1000 steps reduced the risk of neck pain by 14% in those with sedentary jobs.^14^ One possible explanation is that sustaining awkward postures or prolonged sitting during office work increase physical load on body regions, which leads to increased muscle fatigue and discomfort. If there is insufficient time for allowing tissue capacity regeneration, the muscle fatigue may further reduce available capacity. These fatigue‐induced changes are thought to play a role in the pathogenesis of body tissues, leading to neck pain.^28^, ^29^ Increased daily walking steps in sedentary workers may indirectly reflect frequent rest breaks, allowing sufficient tissue recovery to occur. In addition, previous studies have shown that exercise, for example, cycling, can increase blood flow or tissue oxygenation to non‐working or inactive muscles.^30^, ^31^ Thus, it is plausible that lower limb activity, for example, walking, may increase blood flow or oxygenation to neck/shoulder muscles, resulting in a continuous supply of nutrients and the removal of metabolic waste products; thus, speeding up tissue recovery.

The results showed that, after adjusting for confounders, a positive association between BMI and onset of neck pain was found, that is, a higher BMI increased the risk of neck pain. The finding is in line with a recent systematic review showing that a high BMI (>30 kg/m^2^) was a risk factor for incident neck pain in the general population.^32^ One possible explanation for the association between excess body weight and neck pain relates to obesity‐induced low‐grade systemic inflammation.^33^, ^34^ We also found that job security scores, assessed by the Job Content Questionnaire, was negatively associated with onset of neck pain, that is, a higher job security score decreased the risk of neck pain. However, evidence regarding the association between job security and neck pain is still controversial.^35^ Yang et al^35^ conducted a cross‐sectional study using data from the National Health Interview Survey in US population and found that job security was among a set of workplace risk factors associated with neck pain in the past three months. Shan et al^36^ found that job insecurity led to an increased risk of neck pain in Malaysian male workers. On the other hand, Bugajska et al^37^ reported no significant relationship between job security and neck pain in the Polish population. Yang et al^35^ hypothesized that different unemployment benefits in the public welfare system among countries may play a contributing role to the inconsistent findings across studies.

In this study, office workers in both the intervention and control groups who reported neck pain were asked to rate their pain intensity using a 100‐mm visual analogue scale and disability related to neck pain using the NDI. The results indicated no significant difference in pain intensity and disability related to neck pain between the groups. Previous studies showed that there was no significant difference in pain intensity and disability as well as quality of life and health status between those who reported incidence of neck or low back pain in the exercise and control groups.^11^, ^38^ The results of the present study support the notion that effective intervention to prevent neck pain, at least in office workers, may differ from those to alleviate pain intensity and disability level in those with onset neck pain. Neck pain and disability levels among the sample population in this study, who reported the incidence of neck pain, are relatively low. Consequently, we may encounter a floor effect, that is, participants scored at or near the possible lower limit.^39^ Further research should examine the effects of walking intervention in office workers with moderate to high pain intensity or disability to validate the findings of this study.

Four main methodological limitations should be taken into consideration when interpreting the results of this study. First, a convenience sample of office workers from a small number of offices (ie, three government offices and a public university office) was randomly assigned at the cluster level into either the intervention or control groups, which may restrict the external validity of this study. Although the randomization was deemed successful in creating the intervention and control groups with similar baseline characteristics, we found differences in age and duration of employment between the groups at baseline. One reason for such differences is because only four clusters were included in this study, which may introduce sampling errors. However, its impact on the internal validity of the findings is likely to be limited because, based on multivariate logistic regression analysis, the results revealed that age and duration of employment had no significant effect on the incidence of neck pain (Table 3). Also, during the recruitment process of this study, we recorded a lower annual prevalence of neck pain (38%) among office workers who expressed interest in participating in the study, compared to previous studies (between 42% and 69%).^1^, ^2^, ^3^, ^4^ The discrepancy between this study and the previous studies may be due to differences in the definition of neck pain. In this study, only those having neck pain intensity >30 mm on a 100‐mm visual analogue scale were identified as cases, whereas in the previous studies those reporting neck pain, regardless of pain intensity, were identified as cases. Consequently, it is possible that a lower annual prevalence of neck pain among office workers was reported in our study. Caution should be exercised with generalization of the results from this study to other groups of officer workers. Second, no blinding of all participants to treatment allocation was implemented. Participant blinding ensures that the apparent effect (or lack of effect) of treatment is not due to the placebo effect or Hawthorne effect, thus enhancing the internal validity of a study. The influence of the placebo effect or Hawthorne effect on the outcomes of this study, particularly for participants in the intervention group, cannot be excluded. Although blinding participants in an exercise‐related trial is not possible, one strategy that could be used to minimize the expectation bias of participants is to provide a sham intervention to the control group. Third, a number of biopsychosocial factors as well as the diagnosis of nonspecific neck pain were subjective, which poses the risk of the overestimation of exposure in some workers. Researchers should consider the inclusion of objective information from a physical examination to increase data accuracy in future studies. Fourth, we did not assess participants’ daily walking steps at baseline (in both intervention and control groups). Therefore, we did not know that designated daily walking steps calculated by the smartphone application for individuals in the intervention group was higher or lower than their habitual daily walking steps. Future study should be conducted to examine the efficacy of walking intervention to prevent neck pain in those with low habitual daily walking steps relative to the designated daily walking steps calculated by the smartphone application to confirm the present study findings.

## 5 CONCLUSION

A 6‐month prospective, cluster‐randomized‐controlled trial was conducted in a convenience sample of healthy office workers with high risk of neck pain. The results of the present study suggest that the walking intervention can effectively reduce incident neck pain in office workers. The 6‐month incidence of neck pain was reduced by 78% by intervention. However, the walking interventions did not decrease pain intensity and disability in those increasing the number of daily walking steps compared to the control group. Walking with its low cost, easy access, and low impact on musculoskeletal structures is a promising intervention for preventing neck pain in high‐risk office workers.

## DISCLOSURE

*Approval of the research protocol*: The study was approved by the University Human Ethics Committee. *Informed consent*: All participants were given information about the study and were asked to sign a consent form prior to their participation. *Registry and the registration no. of the study/trial*: This trial was registered in the Thai Clinical Trials Registry (TCTR20160928001). *Animal studies*: N/A. *Conflict of interest*: The authors declare no conflict of interests for this article.

## AUTHOR CONTRIBUTIONS

The authors have contributed in the following ways: ES provided the concept/research design, data collection, data analysis, and manuscript writing. RS, PW, and PJ provided the concept/research design, data analysis, and manuscript writing. All authors read and approved the final manuscript.

## References

[1] Cote P , van der Velde G , Cassidy JD , et al. The burden and determinants of neck pain in workers: results of the Bone and Joint Decade 2000–2010 Task Force on Neck Pain and Its Associated Disorders. J Manipulative Physiol Ther. 2009;32(2 suppl):S70‐S86. 19251078 10.1016/j.jmpt.2008.11.012

[2] De Loose V , Burnotte F , Cagnie B , Stevens V , Van Tiggelen D . Prevalence and risk factors of neck pain in military office workers. Mil Med. 2008;173(5):474‐479. 18543569 10.7205/milmed.173.5.474

[3] Hush JM , Michaleff Z , Maher CG , Refshauge K . Individual, physical and psychological risk factors for neck pain in Australian office workers: a 1‐year longitudinal study. Eur Spine J. 2009;18(10):1532‐1540. 19399537 10.1007/s00586-009-1011-z PMC2899383

[4] Janwantanakul P , Pensri P , Jiamjarasrangsri V , Sinsongsook T . Prevalence of self‐reported musculoskeletal symptoms among office workers. Occup Med (Lond). 2008;58(6):436‐438. 18544589 10.1093/occmed/kqn072

[5] Leaver AM , Maher CG , McAuley JH , Jull G , Latimer J , Refshauge KM . People seeking treatment for a new episode of neck pain typically have rapid improvement in symptoms: an observational study. J Physiother. 2013;59(1):31‐37. 23419913 10.1016/S1836-9553(13)70144-9

[6] Sihawong R , Sitthipornvorakul E , Paksaichol A , Janwantanakul P . Predictors for chronic neck and low back pain in office workers: a 1‐year prospective cohort study. J Occup Health. 2016;58(1):16‐24. 26498979 10.1539/joh.15-0168-OA

[7] de Campos TF , Maher CG , Steffens D , Fuller JT , Hancock MJ . Exercise programs may be effective in preventing a new episode of neck pain: a systematic review and meta‐analysis. J Physiother. 2018;64(3):159‐165. 29908853 10.1016/j.jphys.2018.05.003

[8] Hogg‐Johnson S , van der Velde G , Carroll LJ , et al. The burden and determinants of neck pain in the general population: results of the Bone and Joint Decade 2000–2010 Task Force on Neck Pain and Its Associated Disorders. J Manipulative Physiol Ther. 2009;32(2 suppl):S46‐60. 19251074 10.1016/j.jmpt.2008.11.010

[9] Andersen CH , Andersen LL , Pedersen MT , et al. Dose‐response of strengthening exercise for treatment of severe neck pain in women. J Strength Cond Res. 2013;27(12):3322‐3328. 23478473 10.1519/JSC.0b013e31828f12c6

[10] Nikander R , Malkia E , Parkkari J , Heinonen A , Starck H , Ylinen J . Dose‐response relationship of specific training to reduce chronic neck pain and disability. Med Sci Sports Exercise. 2006;38(12):2068‐2074. 10.1249/01.mss.0000229105.16274.4b 17146312

[11] Sihawong R , Janwantanakul P , Jiamjarasrangsi W . Effects of an exercise programme on preventing neck pain among office workers: a 12‐month cluster‐randomised controlled trial. Occup Environ Med. 2014;71(1):63‐70. 24142988 10.1136/oemed-2013-101561

[12] O'Connor SR , Tully MA , Ryan B , et al. Walking exercise for chronic musculoskeletal pain: systematic review and meta‐analysis. Arch Phys Med Rehabil. 2015;96(4): 724–734 25529265 10.1016/j.apmr.2014.12.003

[13] Hurley DA , Tully MA , Lonsdale C , et al. Supervised walking in comparison with fitness training for chronic back pain in physiotherapy: results of the SWIFT single‐blinded randomized controlled trial. Pain. 2015;156(1):131‐147. 25599309 10.1016/j.pain.0000000000000013

[14] Sitthipornvorakul E , Janwantanakul P , Lohsoonthorn V . The effect of daily walking steps on preventing neck and low back pain in sedentary workers: a 1‐year prospective cohort study. Eur Spine J. 2015;24(3):417‐424. 25208502 10.1007/s00586-014-3577-3

[15] Paksaichol A , Janwantanakul P , Lawsirirat C . Development of a neck pain risk score for predicting nonspecific neck pain with disability in office workers: a 1‐year prospective cohort study. J Manipulative Physiol Ther. 2014;37(7):468‐475. 25127997 10.1016/j.jmpt.2014.07.004

[16] Tsauo JY , Jang Y , Du CL , Liang HW . Incidence and risk factors of neck discomfort: a 6‐month sedentary‐worker cohort study. J Occup Rehabil. 2007;17(2):171‐179. 17340188 10.1007/s10926-007-9076-1

[17] Andersen LL , Jørgensen MB , Blangsted AK , Pedersen MT , Hansen EA , Sjøgaard G . A randomized controlled intervention trial to relieve and prevent neck/shoulder pain. Med Sci Sports Exercise. 2008;40(6):983‐990. 10.1249/MSS.0b013e3181676640 18461010

[18] Phakthongsuk P . Construct validity of the Thai version of the job content questionnaire in a large population of heterogeneous occupations. J Med Assoc Thailand. 2009;92(4):564‐572. 19374310

[19] Sitthipornvorakul E , Waongenngarm P , Lohsoonthorn V , Janwantanakul P . Is the number of daily walking steps in sedentary workers affected by age, gender, body mass index, education, and overall energy expenditure? Work. Forthcoming. 10.3233/WOR-203206 32623424

[20] Borghouts JA , Koes BW , Bouter LM . The clinical course and prognostic factors of non‐specific neck pain: a systematic review. Pain. 1998;77(1):1‐13. 9755013 10.1016/S0304-3959(98)00058-X

[21] Kuorinka I , Jonsson B , Kilbom A , et al. Standardised Nordic questionnaires for the analysis of musculoskeletal symptoms. Appl Ergon. 1987;18(3):233‐237. 15676628 10.1016/0003-6870(87)90010-x

[22] Uthaikhup S , Paungmali A , Pirunsan U . Validation of thai versions of the neck disability index and neck pain and disability scale in patients with neck pain. Spine. 2011;36(21):E1415‐E1421. 21228749 10.1097/BRS.0b013e31820e68ac

[23] Rothman KJ , Greenland S . Modern Epidemiology. 2nd ed Philadelphia, PA: Lippincott‐Raven; 1998.

[24] McLean SM , Klaber Moffett JA , Sharp DM , Gardiner E . A randomised controlled trial comparing graded exercise treatment and usual physiotherapy for patients with non‐specific neck pain (the GET UP neck pain trial). Man Ther. 2013;18(3):199‐205. 23085116 10.1016/j.math.2012.09.005

[25] Shnayderman I , Katz‐Leurer M . An aerobic walking programme versus muscle strengthening programme for chronic low back pain: a randomized controlled trial. Clin Rehabil. 2013;27(3):207‐214. 22850802 10.1177/0269215512453353

[26] Sitthipornvorakul E , Klinsophon T , Sihawong R , Janwantanakul P . The effects of walking intervention in patients with chronic low back pain: a meta‐analysis of randomized controlled trials. Musculoskelet Sci Pract. 2018;34:38‐46. 29257996 10.1016/j.msksp.2017.12.003

[27] Lawford BJ , Walters J , Ferrar K . Does walking improve disability status, function, or quality of life in adults with chronic low back pain? A systematic review. Clin Rehabil. 2016;30(6):523‐536. 26088673 10.1177/0269215515590487

[28] Wahlstrom J , Lindegard A , Ahlborg G Jr , Ekman A , Hagberg M . Perceived muscular tension, emotional stress, psychological demands and physical load during VDU work. Int Arch Occup Environ Health. 2003;76(8):584‐590. 12898271 10.1007/s00420-003-0454-5

[29] Martin C , Sun W . Fatigue damage of collagenous tissues: experiment, modeling and simulation studies. J Long Term Eff Med Implants. 2015;25(1‐2):55‐73. 25955007 10.1615/jlongtermeffmedimplants.2015011749 PMC5302026

[30] Andersen LL , Blangsted AK , Nielsen PK , et al. Effect of cycling on oxygenation of relaxed neck/shoulder muscles in women with and without chronic pain. Eur J Appl Physiol. 2010;110(2):389‐394. 20512501 10.1007/s00421-010-1517-4

[31] Tanaka H , Shimizu S , Ohmori F , et al. Increases in blood flow and shear stress to nonworking limbs during incremental exercise. Med Sci Sports Exercise. 2006;38(1):81‐85. 10.1249/01.mss.0000191166.81789.de 16394957

[32] Kim R , Wiest C , Clark K , Cook C , Horn M . Identifying risk factors for first‐episode neck pain: a systematic review. Musculoskeletal Sci Pract. 2018;33:77‐83. 10.1016/j.msksp.2017.11.007 29197234

[33] Roytblat L , Rachinsky M , Fisher A , et al. Raised interleukin‐6 levels in obese patients. Obes Res. 2000;8(9):673‐675. 11225716 10.1038/oby.2000.86

[34] Das U . Is obesity an inflammatory condition? Nutrition. 2001;17(11‐12):953‐966. 11744348 10.1016/s0899-9007(01)00672-4

[35] Yang H , Hitchcock E , Haldeman S , et al. Workplace psychosocial and organizational factors for neck pain in workers in the United States. Am J Ind Med. 2016;59(7):549‐560. 27184340 10.1002/ajim.22602 PMC4979741

[36] Shan CL , Adon MYB , Rahman ABA , Hassan STS , Ismail KB . Prevalence of neck pain and associated factors with personal characteristics, physical workloads and psychosocial among male rubber workers in FELDA settlement Malaysia. Global J Health Sci. 2012;4(1):94‐104. 10.5539/gjhs.v4n1p94 PMC4777017 22980103

[37] Bugajska J , Żołnierczyk‐Zreda D , Jędryka‐Góral A , et al. Psychological factors at work and musculoskeletal disorders: a one year prospective study. Rheumatol Int. 2013;33(12):2975‐2983. 23934521 10.1007/s00296-013-2843-8 PMC3832752

[38] Sihawong R , Janwantanakul P , Jiamjarasrangsi W . A prospective, cluster‐randomized controlled trial of exercise program to prevent low back pain in office workers. Eur Spine J. 2014;23(4):786‐793. 24492949 10.1007/s00586-014-3212-3 PMC3960439

[39] Everitt B . Cambridge Dictionary of Statistics . Cambridge, U.K., New York, NY: Cambridge University Press; 2002.

